# Expanded adipose derived mesenchymal stromal cells are effective in treating chronic insertional patellar tendinopathy: clinical and MRI evaluations of a pilot study

**DOI:** 10.1186/s40634-021-00358-7

**Published:** 2021-07-02

**Authors:** Miguel A. Khoury, Karim Chamari, Montassar Tabben, Khalid Alkhelaifi, Trueba Ricardo, Couto Damián, Pieter D’hooghe

**Affiliations:** 1 Cleveland Orthopedics, Buenos Aires, Argentina; 2 Aspetar Qatar Orthopaedic and Sports Medicine Hospital, P.O. Box 29222, Doha, Qatar; 3 Grupo Médico Rostagno, Buenos Aires, Argentina; 4 Himan Centro de Diagnóstico por Imágenes, Buenos Aires, Argentina

**Keywords:** Cell therapy, Tendon healing, jumper’s knee, Chronic tendinopathy, Patellar tendon

## Abstract

**Purpose:**

Effect of ultrasound guided injections of autologous ASCs in chronic recalcitrant patellar tendinopathy.

**Methods:**

Fourteen patients (16 knees, 12/2 males/females) with chronic, recalcitrant (unsuccessfully treated with nonoperative treatments) insertional PT underwent clinical evaluation and magnetic resonance imaging (MRI) before intervention. Stromal vascular fraction cells (SVF) were expanded by in-vitro culture and characterized by flow cytometry. Players were injected with three bi-weekly injections of ASCs followed by physiotherapy. They underwent serial clinical evaluations during a 12-month period with repeated MRI at 6-month post-injection.

**Results:**

Victorian Institute of sports assessment-patellar tendon questionnaire (VISA-P) scores improved from 43.8 ± 4.9 at baseline to 58.1 ± 7.1, 70.3 ± 7.9 and 78.7 ± 7.5 at 3, 6, and12months follow-up, respectively. (*p* = 0.0004 comparing each variable with the previous one). Mean Visual analogue pain in sports (VAS-sport) score during practice significantly decreased from 7.4 ± 0.5 at baseline to 5.2 ± 1.5 9 (*p* = 0.0005), 3.3 ± 1.1 (*p* = 0.0004) and 1.5 ± 0.7 (*P* = 0.0004) at 3, 6, and 12 months, respectively. Mean Tegner-scores for patients were 8.0 ± 0.8 before injury and 2.3 ± 0.9 before treatment, thereafter, improving to 4.8 ± 0.8 and 7.2 ± 0.7 at 6- and 12- months, respectively (*p* = 0.0001). MRI assessed tendon width’ did not change over the first 6 months post-intervention. Significant changes were observed for: tendon thickness (12.8 ± 1.1 to 10.9 ± 0.7, *P* = 0.0001); tear length (9.3 ± 1.3 to 2.3 ± 0.7, *P* = 0.0001), tear width (6.3 ± 0.8 to 3.4 ± 0.4, *P* = 0.0001), and tear thickness (4.6 ± 0.4 to 2.6 ± 0., *P* = 0.0001) at baseline and 6 months, respectively.

**Conclusion:**

Patients with recalcitrant insertional PT showed significant clinical improvement and structural repair at the patellar insertional tendinopathy after injections of autologous ASCs. Results of this study are promising and open a new biological therapeutic modality to treat PT.

## Introduction

Insertional patellar tendinopathy (PT), commonly known as jumper’s knee, is a highly limiting overuse injury often found in athletes who engage in repetitive forceful loading of the knee extensor apparatus particularly in those with a high volume of training [13].

More specifically, the prevalence of PT has been shown to be highest among sports involving jumping (e.g.volleyball and basketball) and/or involving running and kicking (e.g.soccer and track and field) [25]. Risk factors like gender (males), amount of hours of training, hamstring flexibility and previous knee injury were reported [26]. PT might result in long-lasting symptoms after an athletic career which is an important aspect for the athletes health on the long term [18].

In chronic and recalcitrant cases, surgery might be indicated. In that regard, a randomized study showed that more than half of the patients still had persisting pain at 12 months follow-up post-surgery [3]. The histopathology underlying this condition mainly involves: microinjury of tendon fibers, mucoid degeneration, necrosis, loss of transitional fibrocartilage tissue that attaches to bone generally without inflammation, and increased apoptotic cell death [24].

Healing the tendon and minimizing scar tissue formation, recovering structural properties and reinforcing tensile strength is the goal of biological treatments [26]. By definition, stromal/mesenchymal cells are able to self-renew and exist in every human tissue in an undifferentiated or unspecialized state. These cells are capable of differentiating or specializing along multiple lineages [2, 4], and adipose tissue is an excellent source of adipose derived mesenchymal stromal cells (ASCs). These have been receiving increasing attention over the years toward enhancing tendon healing. Evidences from in vitro and in vivo studies suggest that ASCs can contribute to accelerate and improve the quality of healed tendons [28].

We hypothesized that ultrasound guided injections of autologous ASCs in chronic recalcitrant patellar tendinopathy may be useful in healing the damaged tendon and further decreasing the need of surgery and a successful return to play. We also aimed at assessing the role of MRI evaluation in this pathology and intrinsically evaluate the safety of ASCs culture preparation as an alternative treatment of PT.

## Material and methods

This prospective longitudinal case series exploratory study was approved by the ethical committee of Regenerar^R^ laboratory (Clinical Trial MR00124). Patients with patellar tendinopathy who referred to our institution from January 2015 to February 2019 were invited to participate giving signed informed consent. Before intervention, a detailed clinical history was performed and visual analog scale (VAS) for maximun pain in sports participation [12], Knee Victorian Institute of sports assessment-patellar tendon questionnaire (Visa-P) [38], Tegner-score before symptoms onset and pre-treatment [6] X-rays and MRI evaluations of the concerned knee were performed. The diagnosis of patellar tendinopathy was confirmed using MRI standard imaging protocol. The intervention consisted in three bi-weekly doses of 8 × 0^6^ ASCs. The follow-up protocol (Table 1) included VAS-sport [12]. Visa-P questionnaire, patient satisfaction and adverse events at 3, 6 and 12 months. MRI was repeated at 6 months and Tegner-score at 6 and 12 months. Minimum clinically important difference (MCID) were considered present when a difference of 13 points in Visa-P [38], 2 points in VAS-sport [12] and 1 point in Tegner [38] were identified. Patient satisfaction was evaluated using a scale including the following categories: improved symptoms, no change-, and worse-symptoms during the practice of sports.

**Table 1:** Sample description

Patient	Side	Sex	Age	BMI	Symp. Month	Hour/ Week	Sport	Previous Treatment	MRI
1		F	24	21.2	12	10	Running	Polidocanol	BME-IFPE
2		F	38	22.3	24	12	Running	Corticosteroids -ESWT	BME-IFPE
3		M	18	23.4	18	11	Running	PRP	BME
4		M	35	25.1	18	11	Running	PRP-ESWT	–
5		M	28	24.4	11	12	Soccer	Prolotherapy	–
6		M	41	25.3	30	12	Soccer	PRP	BME-IFPE
7		M	43	24.7	21	12	Soccer	Corticosteroids -Polidocanol	–
8		M	31	24.3	24	9	Volley	PRP	IFPE
9		M	46	23.7	24	9	Volley	ESWT	IFPE
10		M	48	23.8	19	11	Handball	ESWT	BME
11		M	37	23	16	9	Athletic	PRP-ESWT	–
12		M	45	24.1	13	8	Basketball	Prolotherapy- PRP	–
13	Bilateral	M	23	23.1	25	11	Basketball	Prolotherapy	–
14	Bilateral	M	27	24.1	13	8	Running	PRP	Bilateral BME
15		M	43	25.3	36	7	Tennis	PRP	–
16		M	45	26.7	24	5	Soccer	ESWT	IFPE

*BME* Bone Marrow Edema, *IFPE* Infrapatellar Fat Pad Edema, *PRP* Platelet Rich Plasma, *ESWT* Extracorporeal Shock Wave Therapy

### Deleted

#### Participants

A total of 16 patients (males = 14; females = 2) were initially recruited (Table 1). They all presented the following standard physician established diagnostic criteria: (i) history of exercise-related pain located at the PT insertion for at least 6 months, (ii) tenderness to palpation of the tendon substance and a positive result from an MRI (thickening and tear of the patellar tendon). Patients had to have already unsuccessfully attempted to treat their conditions with exercise-based rehabilitation and different treatment modalities for a minimum of 6 months. Patients were excluded if they had a history of knee or patellar tendon surgery or any inflammatory or prominent degenerative joint condition affecting the knee, contraindication to injection therapy, bleeding diathesis or being on anti-coagulants, local or systemic infection, injections of corticosteroid medication in the past 3 months. All participants were classified as having a degree 3B Blazina classification [5]. Two male patients (number 15 and 16 in Table 1) withdrew consent 3 and 6 months after injections and had surgical treatment. These two patients had a baseline scores of 44 and 45 in VISA-P, 7.5 and 7.2 in VAS-Sp, 2 and 1 in Tegner scores with no improvements at 3 months. All remaining 14 patients were followed during the 12 months’ period.

No patients have previously received mesenchymal cells due to any medical condition.

### Intervention

#### Collection, Characterization and Isolation of Adipose Tissue

This intervention has been performed according to validated procedures already published [19]. Briefly ASCs were stained with antibodies against CD73, CD90, CD105, CD45, CD34, CD11b, CD19 or CD79a, CD14, and HLA class II, to determine surfer marker expression. In accordance with the International Society for Cellular Therapy (ISCT) recommendations: ≥ 95% of the MSC population expressed CD105, CD73 and CD90, as measured by flow cytometry. Additionally, these cells lacked expression (≤ 2% positive) of CD45, CD34, CD14 orCD11b, CD79a or CD19 and HLA-DR (class II.20) Also, the cells were able to differentiate to osteoblasts, adipocytes and chondroblasts under standard in vitro differentiating conditions [14]. FACS Canto II BD flow cytometer instrument was used for running samples. The samples were immunostained with a determined panel of monoclonal antibodies (mentioned above) conjugated with different fluorochromes and mouse isotype antibodies were used as control. The results were analyzed with the software Infinicyt version 1.7i.

### Percutaneous ultrasound guided intra-lesional delivery of ASCs

**Fig. 1:**
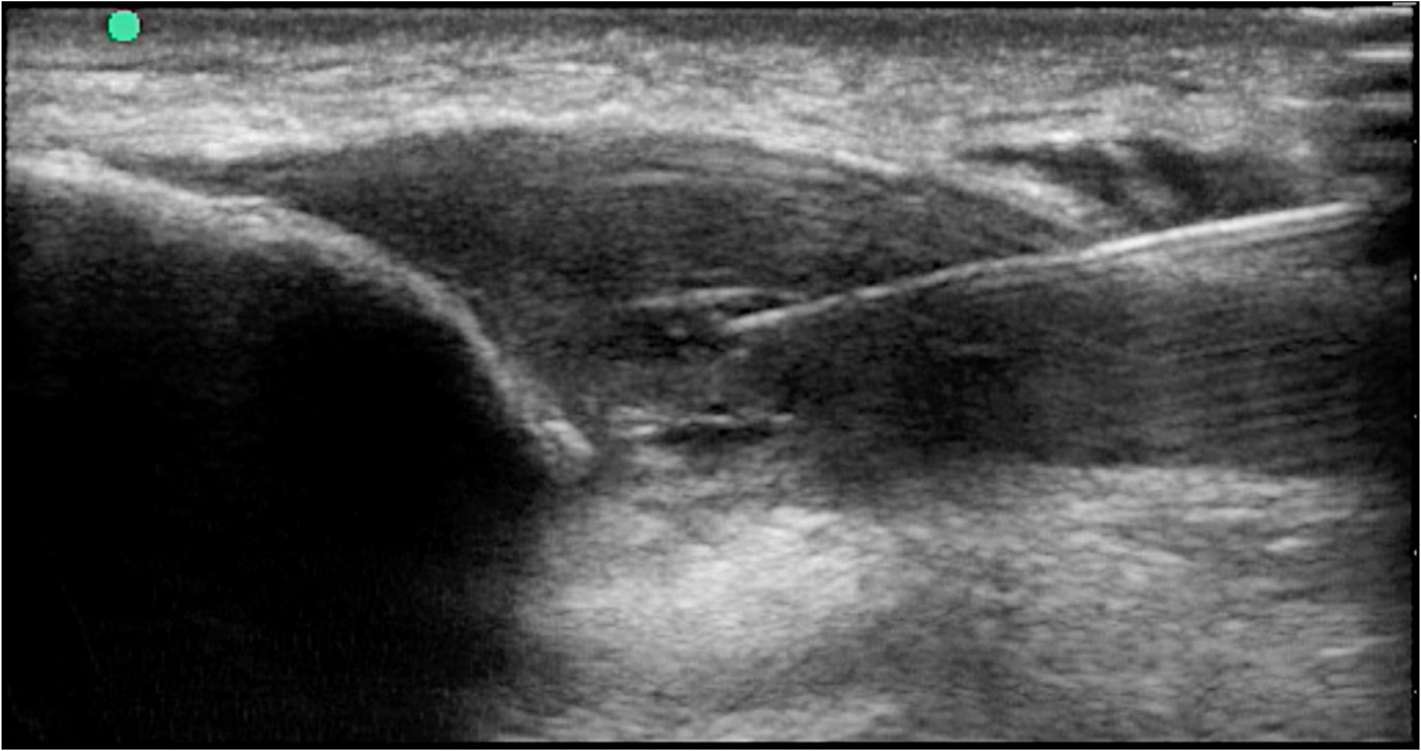
Ultrasound guided cell injection into the hypoechoic area of the proximal patellar tendon

Autologous ASCs were injected at an average of 1 month after tissue collection. The cultured ASCs were washed twice and suspended in 3 ml of HBSS (Gibco, Cat # 14025088). Prior to the injection, the skin was prepared by aseptic technique with iodine solution. An average of 6 × 10^6^ ASCs were injected 3 times separated by two weeks in each of the affected tendon. The cells were delivered percutaneously under local anesthesia (2% lidocaine was previously superficially injected to numb the skin). An ultrasound equipment (Esaote X6, L 4–15 mHz probe) was used to guide injections which were performed by the same physician (MAK). the injection was performed deep into the tendon substance reaching t the hypoechoic area (Fig. 1).

### Postprocedure protocol

Immediately after the injection, the patient was kept in a seated position without moving the legs for 15 min. Patients were sent home with instructions to rest for approximately 24 h before performing any physical activity. If necessary, patients were allowed to use acetaminophen 500 mg every 6 h. Only 5 patients used it during the first 48 h post-injection. Use of nonsteroidal anti-inflammatory medication was not allowed. After 48 h, patients were prescribed an initial standardized rehabilitation protocol to follow for 2 weeks under the supervision of a physiotherapist. A formal eccentric muscle- and tendon-strengthening program was initiated after this program. At 2 months after the third injection, patients were clinically evaluated to determine the proceeding with progression of the successive individualized rehabilitation or recreational activities as part of the protocol. Patellar tendinopathy was treated by application of eccentric exercise with the use of a decline board. The decline enables better isolation of the knee extensor mechanism in squat exercises [32].

#### Magnetic resonance imaging

The equipment used was a 1.5 T MRI scanner (Philips, Eindhoven, The Netherlands) Flex-M Coil. An 8-channel coil was used with patients in the supine position and the affected leg extended. STIR Coronal, Axial & Sagital view (FOV 130 mm, TR 3500, TE 50, TI 100) Cut thickness 3 mm. The evaluation included the extent of tendinosis and tendon tear. The radiological interpretation was performed by two experienced sports trauma radiologists [15].

**Fig. 2:**
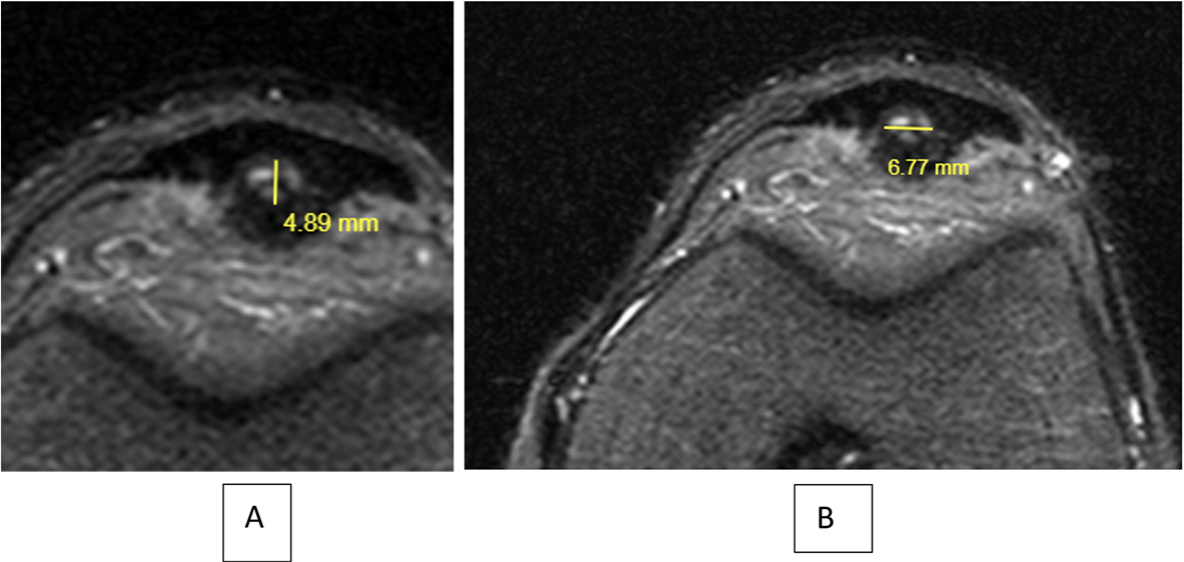
**A** MRI evaluation: Thickness of the tear. **B** Width of the tear

Images were reviewed on our institutional picture archiving and communication system (PACS). An electronic caliper tool in PACS was used to determine patellar tendon measurements; therefore, all measurements taken were automatically standardized. Tendon dimensions were measured with sagittal or axial (anterior to posterior) views: (A) thickness (anterior to posterior) of the patellar tendon on the first axial slice below the inferior patellar bone. (B), width (medial to lateral) of the patellar tendon, (C) length of the tear, (D) thickness of the tear and (Fig. 2 A); (E) width of the tear (Fig. 1B). The tear length dimension (C), were measured with sagittal MRI scans that showed maximum dimension of the tear. The thickness and width dimensions (D and E) were measured in the axial view that maximally showed each dimension, which corresponded to 1 slice below the inferior pole of the patella.

#### Statistics

Data were processed with Stata 14.1 software (StataCorp, Texas, USA). Values are displayed as mean ± standard deviation (SD) or median and range, according to the variable distribution. Normality of numerical variables was verified using the Wilk-Shapiro test. To compare values at two different times, Paired t-test or Wilcoxon sign rank test were used if variables were normally distributed or not, respectively. *P* < 0.05 were considered statistically significant.

## Results

A total of 16 knees (7 left, 9 right) from 14 patients (12 male, 2 female) with a mean age of 34.6 ± 9.5 years were prospectively evaluated. Mean BMI was 23.7 ± 1.1 kg/m^2^, median time of Chronic Insertional Patellar Tendinopathy symptoms was 18 (range 11–30) months and median of training time, 11 h/week (range 8–12).

### Clinical results

Clinical evaluations are presented in Table 2.

**Table 2:** Clinical evaluation

	Pretreatment	3 months	6 months	12 months
Knee Visa-P score	43.8 ± 4.9	58.1 ± 7.1*	70.3 ± 7.9*	78.7 ± 7.5*
Pain (VAS-Sport)	7.4 ± 0.5	5.2 ± 1.5 9^*^	3.3 ± 1.1*	1.5 ± 0.7^*^
Tegner-score	2.3 ± 0.9	–	4.8 ± 0.8*	7.2 ± 0.7*

*Statistically significant differences comparing each value with the previous one (*p* < 0.001)

**Fig. 3:**
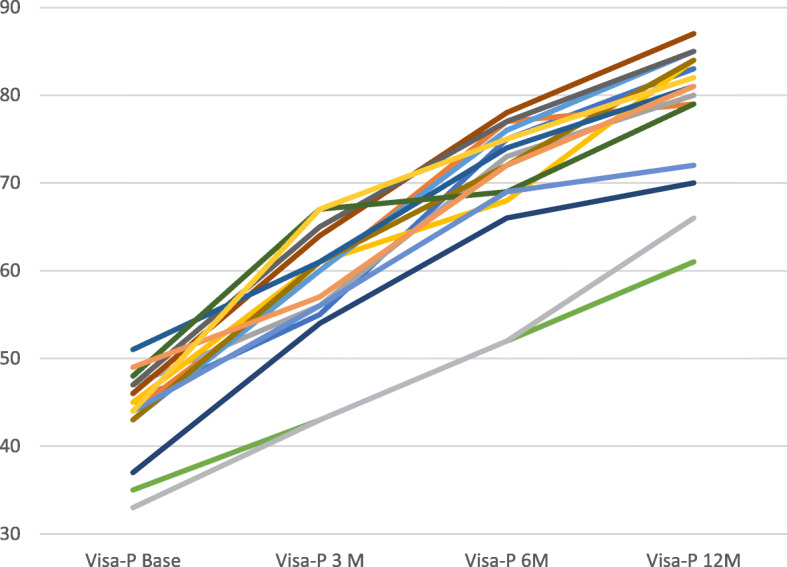
Individual Knee Victorian Institute of sports assessment-patellar tendon questionnaire (Visa-P) scores. Baseline, 3, 6 and 12 months

VISA-P significantly improved at 3, 6 and 12 months (Table 3) compared to pre-intervention (*p* < 0.001). Figure 3 shows individual Knee Visa-P scores. Baseline, 3, 6 and 12 months.

**Table 3:** MRI Outcomes (expressed in mm)

MRI	Baseline Before treatment	6 Months	*P* value
Tendon-width	30.8 ± 2.6	30.7 ± 2.5	0.3724
Tendon-thickness	12.8 ± 1.1	10.9 ± 0.7	0.0004
Tear- length	9.3 ± 1.3	2.3 ± 0.7	0.0004
Tear- width	6.3 ± 0.8	3.4 ± 0.4	0.0004
Tear thickness	4.6 ± 0.4	2.6 ± 0.7	0.0004

Visa-P score minimum clinically important difference (MCID) of 13 points compared to the baseline were present in 12 cases at 3 months and in all the 16 cases at 6 months.

**Fig. 4:**
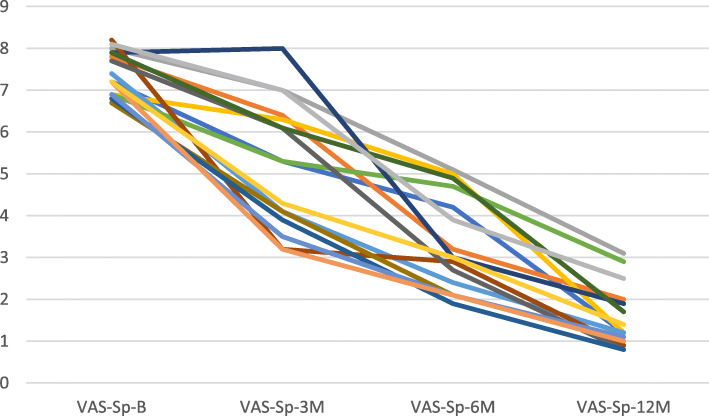
Individual knee Visual analogue scale sports (VAS-Sp) at baseline (VAS-Sp-B), 3 months (VAS-Sp-3 M), 6 months (VAS-Sp-6 M) and 12 months (VAS-Sp-12 M)

Mean Pain during practice (VAS-Sport) score significantly improved at 3, 6, and 12 months (Table 3) compared to pre-intervention (*p* < 0.001). Figure 4 shows individual Knee VAS-Sport at Baseline, 3, 6, and 12 months.

VAS-Sp minimum clinically important difference (MCID) of 2 points compared to baseline were present in 7 cases at 3 months, in 15 cases at 6 months and in the all the 16 cases at 12 months.

**Fig. 5:**
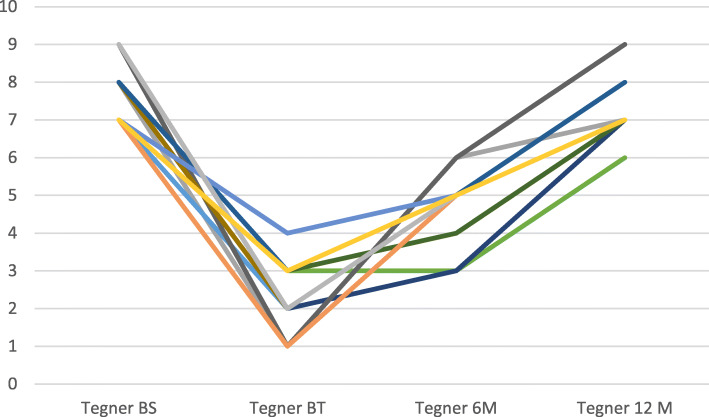
Individual patient Tegner Score: BS: Before symptoms, BT: Before treatment, 6 M: 6 months post-treatment and 12 M: 12 months post-treatment

Mean Tegner-scores for patients were of 8.0 ± 0.8 before injury and 2.3 ± 0.9 before treatment (*p* < 0.0001). Tegner-score significantly improved at 6 and 12 months compared (Table 3) to pre-intervention (*p* < 0.001). Figure 5 shows individual Tegner-score values before injury, before treatment (Baseline) and at 6 and 12 months post-treatment.

Compared to Tegner-score before treatment, MCID of 1-point improvement was observed in 15 cases at 6 months and in all the 16 cases at 12 months based. In 7 cases, at 12 months, the Tegner scores returned to pre-injury values.

### MRI score evaluation

MRI evaluation scores are shown in Table 3.

**Fig. 6:**
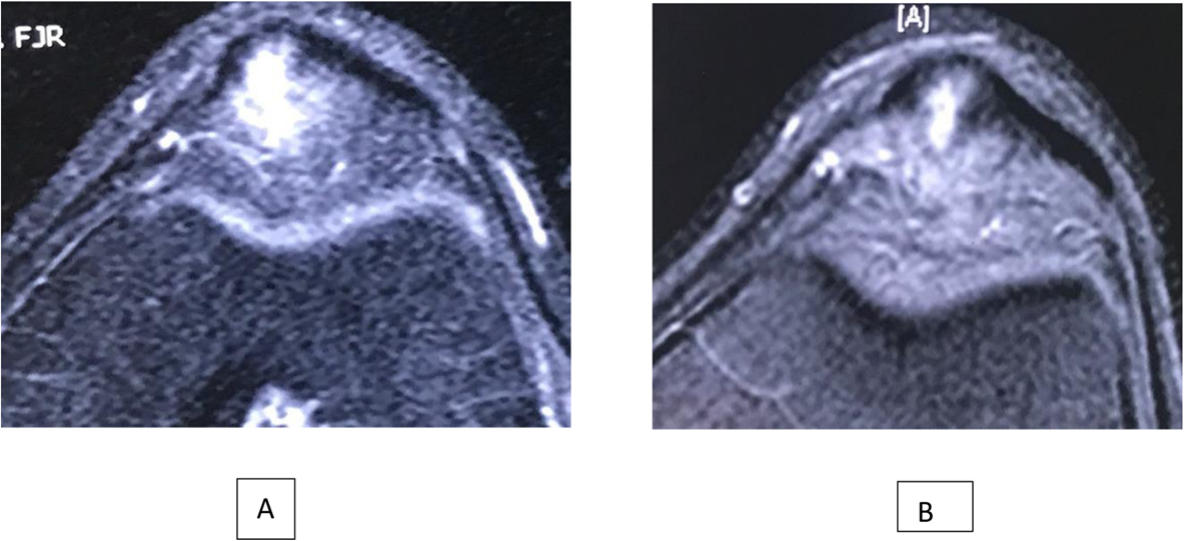
**A**: Axial STIR MR image of the tear of the patellar tendon pre-injections or baseline. **B**: MRI axial view of the patellar tendon at 6 months post-injections

**Fig. 7:**
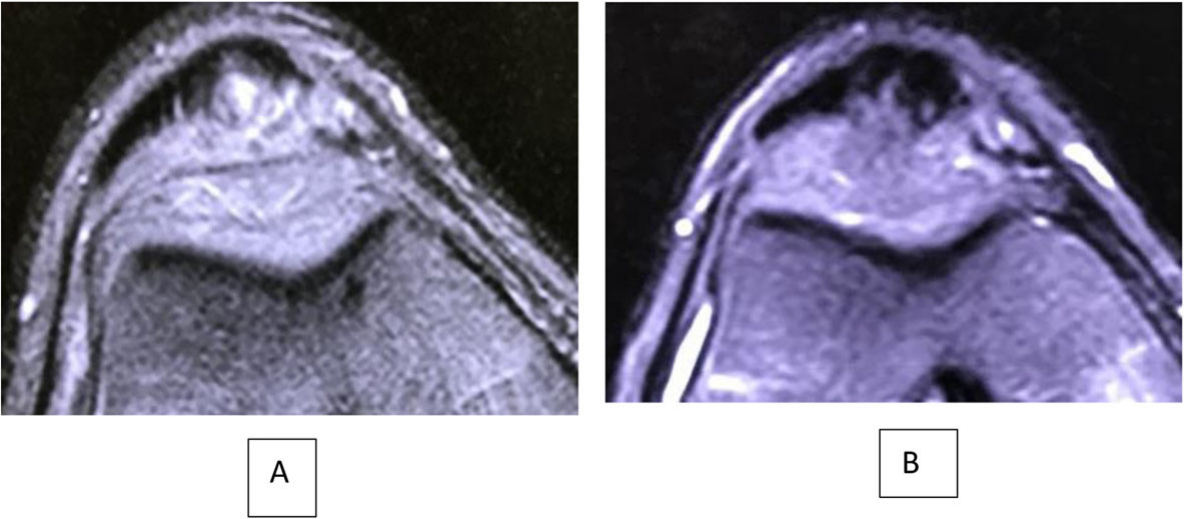
**A** Pre-injection axial STIR MR image showing injury of the patellar tendon and **B**: 6 months Post-injections axial view MRI showing partial healing of the tendon

**Fig. 8:**
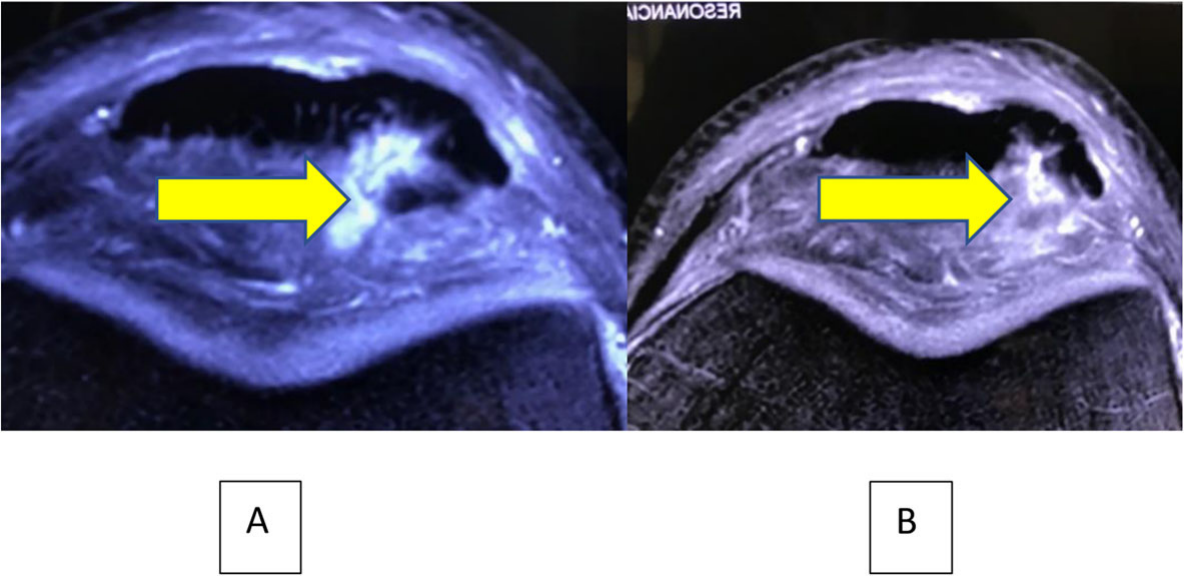
**A** Pre-injection axial STIR MR image showing injury of the patellar tendon and **B**: 6 months Post-injections axial view MRI showing partial healing of the tendon. Observe Fat Pad Edema decreasing over a 6 months period. Yellow arrow

**Fig. 9:**
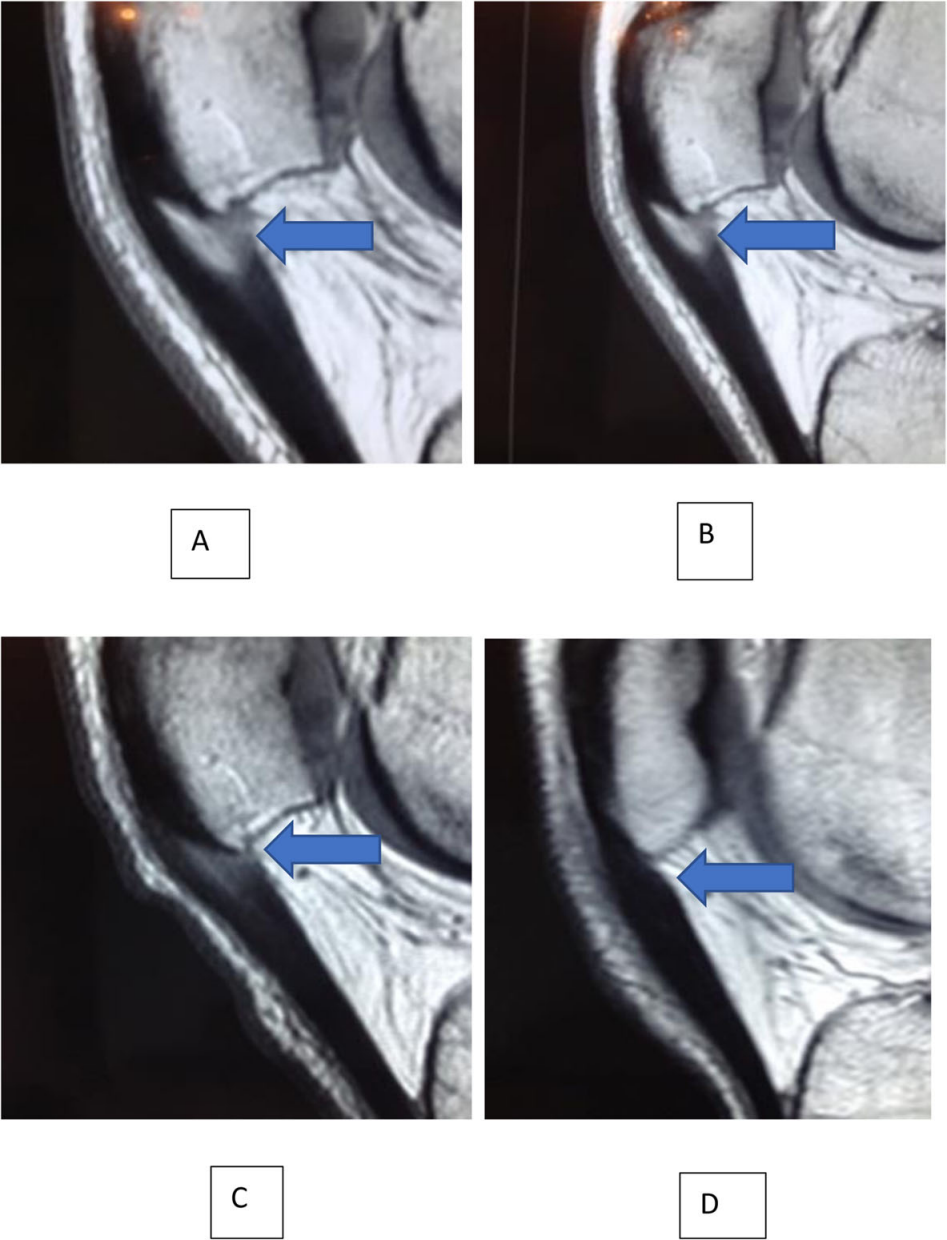
Sagittal T1 MR images of insertional tendinopathy healing at (**A**) Baseline, (**B**), 3, (**C**), 6, and (**D**), 12 months post-injections. Note the decreasing area of tendinosis\Tear (Blue Arrows). Image D: Only a thickening of the tendon can be observed

In pre-treatment MRI, 7 knees had Bone marrow edema (BME) of the inferior pole of the patella, 5 knees had infrapatellar fat pad edema (IFPE) and 3 knees had combined BME and IFPE. Progression of healing of the patellar tendon was observed in all knees. Figs. 6, 7, 8, 9.

### Patient satisfaction

Of the 14 patients (16 knees), at 3 months’ post-injection, 9 patients were improved, 4 patients reported no change and 1 patient as being worse. At 6 months’ evaluation, 1 patient reported disappearance of symptoms, 10 patients improved symptoms and 3 with no change. At 12 months, 9 patients were reported disappearance of symptoms, and 7 with improved symptoms. Thirteen of the patients were ready to undergo the procedure again if needed and 13 of 14 patients would recommend this procedure to other athletes.

### Adverse events

One subcutaneous haematoma and further induration were observed in the site of tissue harvest. It resolved without further treatment. No other complications were observed. No residual symptoms or disability in the periumbilical zone was observed. There were no infections, or other untoward effects.

## Discussion

This study showed that 3 injections of ultrasound guided ASCs were effective in decreasing pain and improving functional and structural repair during the 12 months follow-up of patients with chronic recalcitrant insertional PT. In addition, this study showed that these injections were safe. The structural repair of the tendons was evidenced by MRI at 6-months.

Current PT treatments include exercises, load management and biomechanical interventions [1]. Placebo-controlled studies including shockwave therapy [22], corticosteroid [21], sclerosing injections with polidocanol [17], and Leucocyte rich or Leucocyte poor platelet plasma [35] have been reported with different degrees of effectiveness yet leaving a substantial number of patients with persisting symptoms [17, 21, 22].

The present study supports the promising results using cell therapy in previous manuscripts. Pascual Garrido et.al. showed statistically clinical improvement at 5-year follow-up with non-expanded BM-MNCs (Bone Marrow Mononuclear cells) inoculation for the treatment of chronic patellar tendinopathy [30]. Although the optimal cell number to treat this condition is not known, a potential advantage of our procedure was that we were able to implant a higher number of mesenchymal stromal cells compared to the number of stromal cells provided by BMAC procedures, that yields less than 1% of mesenchymal stromal cells per procedure [36]. Clarke et al. [9] using ultrasound-guided injection of expanded autologous skin-derived tendon-like cells concluded that they can be safely used to treat patellar tendinopathy with, in the short term, faster response of treatment and significantly greater improvement in pain and function than injection of plasma alone. The use of ASCs to treat lateral elbow tendinopathy with promising results has also been reported by our group [19].

It has been previously shown that the responsiveness of the VISA-P scale for patellar tendinopathy in athletes who showed an absolute change greater than 13 points in the VISA-P score (or 15.4–27% of relative change) achieved a 98% probability of a minimal important change (MICD) in their status. In contrast there was only a 45% probability of change when minimum clinically important difference (MICD) was not achieved [16]. In our study, we used the MCID of the VISA-P, to determine whether such a difference is present over time and MICD was obtained during the 12 months follow-up evaluation. The improvement for successful treatments in the VISA-P score from baseline to the 12 months follow-up was 34,9 points. MCID of 13 points compared to pre-intervention were present in 12 knees at 3 months and in all the 16 knees at 6 months. In our study 14 out of the 16 knees had 64 points or greater at 6 months post-injection with the observed clinical improvement. It has been also shown that symptomatic, players with insertional patellar tendinopathy are still able to play with Visa-p score of around 64 points [25]. Taking all this information together we can assume that our results are effective in allowing players to return to play.

Surgical treatment has been recommended when nonoperative treatment fails. In a review, Coleman et al. [10] reported in a literature review, that the success rates ranged from 54% to 100% after open patellar tenotomy, with an “overall” success rate of 83%. However, they also found that the quality of the studies was generally low. In contrast to these findings in a randomized study, Bahr et. Al. [3] concluded that surgical treatment and eccentric strength training can produce significant improvement in terms of pain and function scores. It appears that about only 50% of all patients will be able to return to sport within one year after treatment with each option, and fewer still will have relief of all symptoms. In the absence of other validated treatment options, they suggested that eccentric training, a low-risk and low-cost option, should be tried before surgery is considered [3]. This information also suggests that operative procedures in athlete’s tendons is not always effective. We believe that ASCs injection is justified before considering surgical treatment for patellar tendinopathy. Future studies are needed to (i) report on the cases where ASCs injections would result ineffective, and surgery would be needed and (ii) if ASCs injections would affect future surgical results.

Golman et.al [15]. showed that 91% of PPTTs (Partial Patellar Tendon Tears) were found in the posterior and posteromedial portion of the tendon in patients with patellar tendinopathy. The most sensitive predictor for the presence of a partial tendon tear in their study was the thickness of the tendon, in which thickness > 8.8 mm was strongly correlated with the presence of a tear. In the present study patients’ mean thickness was 12.8 ± 1.1 mm, and in agreement to the latter study we found in all the patients a tendon tear [15]. On MRI images we also found that there was an apparent delay between the observed clinical improvement and the structural repair as shown in the 6 months post-injection. MRI also allowed us to evaluate associated finding to insertional patellar tendinopathy. Ward et al. [39] hypothesized that fat pads contribute to the pathogenesis of tendinopathy by producing and delivering cytokines that drive the pathophysiology. The densely vascularized fat pad is the ideal origin for blood vessels infiltrating the tendon. These vessels deliver the cytokines produced in the fat pad to the tendon. In accordance with these findings, Ogon et al. [27] identified prognostic outcome factors in arthroscopic treatment of chronic PT. They found that preoperative infrapatellar fat pad edema (IFPE) alone or simultaneous bone marrow edema (BME) on preoperative MRI were associated with inferior functional outcome and delayed return to sports. Knowledge about these predictive factors might improve risk stratification, individualize treatment and postoperative rehabilitation, and contribute to improve clinical outcome. Moreover, current findings offer the potential for novel therapeutic approaches. In our study, no clear relationship between these associated MRI findings and outcome could be established. Patellar tendinopathy is frequently accompanied by changes in ultrasound and magnetic resonance imaging (MRI), such as tendon thickening, signal intensity changes and tears of the tendon, these changes could also occur in asymptomatic athletes [27]. The clear relationship between symptoms and pain in imaging studies is not well stablished [16].

Biological treatment of chronic patellar tendinopathy is challenging, mainly due to the low healing capacity of the tendons. This results in long and tedious therapies, therefore, identifying alternative strategies is a priority [1]. The tendon itself is relatively poor in cell number, with a low turnover rate [11]. The existing traditional view that focuses on the multipotent differentiation capacity of these cells should be expanded to include their equally interesting role as cellular modulators that brings them into a broader therapeutic scenario. These trophic and immunomodulatory activities suggest that MSCs may serve as site-regulated “drugstores” in vivo [7, 8]. The potential mechanisms of action proposed for MSCs are related to secretion of various cytokines and growth factors to adjacent cells, mainly recruiting macrophages and cells from the endothelial lineage. This is known as paracrine effect which may elicit cellular proliferation and damaged tissue healing [8]. A second mechanism would be through immunomodulatory properties that could improve healing of injured tissues, through inhibiting T-cell proliferation and further affecting IL-2, IL-10 signaling [33]. In spite of the little evidence for in vivo proliferation of delivered cells due to strong cell-cell contact inhibition, lack of a ready mesenchymal cell-friendly niche and the lower atmospheric oxygen at the recipient tissue, a third potential mechanism could be related to the differentiation into targeted cell types and further contribution to wound repair [34].

The limitations of the present study are related to (i) the small number of knees involved (ii) the lack of a control group and (iii) the outcome assessors that were not blinded. One of the main concerns about safety of stromal cells is the potential for carcinogenesis. Previously reported experiences found no evidence of MSCs related tumor growth in more than 1000 patients treated for several conditions. Therefore, it looks like carcinogenic potential of MSCs might not be as high as previously thought [20]. Recently, the Food and Drug Administration (USA) warned of serious potential risks to patients from “stem cell therapies” administered for uses other than hematopoietic or immunologic reconstitution. Infections were also reported after reception of bacterially contaminated umbilical cord blood–derived stem cell products, intrathecal or intravitreally injections. In contrast to these reports, it is important to state that in cases of stem cell administration for tendon healing, adverse effects have not been reported until present [37]. In spite of this findings there is plenty of evidence based on the current clinical trials, that cultured MSCs therapy rather appears to be safe [23, 31]. In the present study there was only one patient with a mild hematoma and induration in the site of the obtention of the adipose tissue with spontaneous resolution In 4 days. Two patients withdrew consent at 3 and 6 months post-injections, and had surgery. We consider this as a ASCs treatment failure in this series. These patients presented among the largest BMI and Symptoms duration before intervention. This raises the question whether these variables would represent risk factors to intend a conservative treatment, which deserves appropriate research studies.

## Conclusion

Despite the fact that two cases failed in this small series, treating chronic recalcitrant insertional PT by using expanded autologous ASCs injection in weight bearing sports’ athletes demonstrated that the intervention was efficacious and safe in improving pain and performance until a 12-month follow-up period. Structural repair was also already significant at 6 months. In spite of the low level of evidence for use of stromal cells [29], this pilot study supports that 3 bi-weekly ASCs injections could be considered as a promising way to reverse the effects of degenerative process in chronic insertional PT. In the future well-designed, prospective randomized controlled trials are necessary to define the role of cell therapy in treating insertional PT.

## Data Availability

Available.

## Abbreviations

ASCs: Adipose derived Stromal Cells
BM-MNCs: Bone marrow Mononuclear cells
BME: Bone marrow edema
ET: Eccentric Training
IFPE: Infrapatellar fat pad edema
MSCs: Mesenchymal Stromal Cells
MRI: Magnetic Resonance Imaging
MR: Magnetic Resonance
MCID: Minimal clinically Important difference
PRP: Platelet Rich Plasma
PT: Patellar Tendinopathy (insertional)
PPTTs: Partial Patellar Tendon Tears
SVF: Stromal vascular fraction
VAS: Visual Analog Scale
Visa-P: Victorian Institute of sports assessment-patellar tendon questionnaire
